# Integration of structural MRI and epigenetic analyses hint at linked cellular defects of the subventricular zone and insular cortex in autism: Findings from a case study

**DOI:** 10.3389/fnins.2022.1023665

**Published:** 2023-02-03

**Authors:** Emi Takahashi, Nina Allan, Rafael Peres, Alpen Ortug, Andre J. W. van der Kouwe, Briana Valli, Elizabeth Ethier, Jacob Levman, Nicole Baumer, Keita Tsujimura, Nauru Idalia Vargas-Maya, Trevor A. McCracken, Rosa Lee, Alika K. Maunakea

**Affiliations:** ^1^Department of Radiology, Athinoula A. Martinos Center for Biomedical Research, Massachusetts General Hospital and Harvard Medical School, Charlestown, MA, United States; ^2^Department of Radiology, Massachusetts General Hospital and Harvard Medical School, Boston, MA, United States; ^3^Epigenomics Research Program, Department of Anatomy, Institute for Biogenesis Research, Biochemistry and Physiology, John A. Burns School of Medicine, University of Hawai’i at Mānoa, Honolulu, HI, United States; ^4^Department of Behavioral Neuroscience, Northeastern University, Boston, MA, United States; ^5^Department of Mathematics, Statistics and Computer Science, St. Francis Xavier University, Antigonish, NS, Canada; ^6^Department of Neurology, Boston Children’s Hospital and Harvard Medical School, Boston, MA, United States

**Keywords:** epigenetic, MRI, SVZ, cortex, autism, DNA methylation

## Abstract

**Introduction:**

Autism Spectrum Disorder (ASD) is a neurodevelopmental disorder characterized by deficits in social interaction, communication and repetitive, restrictive behaviors, features supported by cortical activity. Given the importance of the subventricular zone (SVZ) of the lateral ventrical to cortical development, we compared molecular, cellular, and structural differences in the SVZ and linked cortical regions in specimens of ASD cases and sex and age-matched unaffected brain.

**Methods:**

We used magnetic resonance imaging (MRI) and diffusion tractography on *ex vivo* postmortem brain samples, which we further analyzed by Whole Genome Bisulfite Sequencing (WGBS), Flow Cytometry, and RT qPCR.

**Results:**

Through MRI, we observed decreased tractography pathways from the dorsal SVZ, increased pathways from the posterior ventral SVZ to the insular cortex, and variable cortical thickness within the insular cortex in ASD diagnosed case relative to unaffected controls. Long-range tractography pathways from and to the insula were also reduced in the ASD case. FACS-based cell sorting revealed an increased population of proliferating cells in the SVZ of ASD case relative to the unaffected control. Targeted qPCR assays of SVZ tissue demonstrated significantly reduced expression levels of genes involved in differentiation and migration of neurons in ASD relative to the control counterpart. Finally, using genome-wide DNA methylation analyses, we identified 19 genes relevant to neurological development, function, and disease, 7 of which have not previously been described in ASD, that were significantly differentially methylated in autistic SVZ and insula specimens.

**Conclusion:**

These findings suggest a hypothesis that epigenetic changes during neurodevelopment alter the trajectory of proliferation, migration, and differentiation in the SVZ, impacting cortical structure and function and resulting in ASD phenotypes.

## Introduction

Autism Spectrum Disorder (ASD) is a neurodevelopmental disorder characterized by deficits in social interaction and communication and repetitive, restrictive behaviors (American Psychiatric Association, 2013; de la Torre-Ubieta et al., 2016). Intellectual disability and language deficits are often observed, as well as several different comorbidities such as epilepsy, requiring varying needs of support and treatment (American Psychiatric Association, 2013). Current prevalence rates of ASD are estimated to be over 1/100 in the global population and 1 in 68 children in the United States (Developmental Disabilities Monitoring Network Surveillance Year 2010 Principal Investigators; Centers for Disease Control and Prevention (CDC), 2014), due to increasing awareness, expanded diagnostic criteria, and the development of more accurate diagnostic instruments (Hodges et al., 2020; Takumi et al., 2020; Zeidan et al., 2022). Studies in monozygotic (MZ) and dizygotic (DZ) twins showed ASD concordances of 60–92% for MZ twins and 0–36% for DZ twins (Bailey et al., 1995; Ronald and Hoekstra, 2011). Furthermore, in families with two children with ASD, the probability of the third-born male child being affected with ASD is 50% (Zhao et al., 2007). Approximately 40% of the ASD cases have a known genetic component, which can be inherited or acquired *de novo*. Most of the genes implicated in the ASD etiology fall into two main categories: genes encoding proteins involved in synapse development and function, and genes encoding proteins related with chromatin remodeling (Tremblay and Jiang, 2019). Some of the best studied ASD related genes include SHANK3, FMR1, and MECP2 (Perez-Garcia and O’Leary, 2016; Varghese et al., 2017; Balaan et al., 2019; Grasselli et al., 2020, p. 3). However, most cases of ASD do not have known genetic causes and are referred to as idiopathic (Varghese et al., 2017).

In humans, the subventricular zone (SVZ) is a highly proliferative and heterogeneous region that contributes to neurogenesis and gliogenesis during gestational stages and contributes to the expansion of the cerebral cortex (Luskin and Coskun, 2002; Zecevic et al., 2005; Wu et al., 2014). At this point of development the structure of the SVZ is evident, and its function as one of two known sources of adult neurogenesis is established (Eriksson et al., 1998). In adults, the SVZ is directly adjacent to the wall of the lateral ventricle, just above the striatum and is organized into four distinct layers: a single cell thick layer of ependymal cells that form an epithelium between the SVZ and the wall of the lateral ventricle; the hypocellular gap, which mostly contains expansions of ependymal cells and processes of astrocytes with very few cell bodies present; a “ribbon” of GFAP + astrocyte cell bodies of varied morphologies, processes, and orientation; and the transitional layer that serves as the border between the SVZ and the adjacent striatal brain parenchyma (Sanai et al., 2004, 2011; Quiñones-Hinojosa et al., 2006; Kotagiri et al., 2014). During the first 6 months of life, there is a decline in SVZ neurogenesis, and after 18 months, proliferative activity and the number of immature neurons reach the trace levels seen in adulthood. Markers of proliferation, including Ki-67 and bromodeoxyuridine (BrdU) have been observed to be expressed by cells within the SVZ, which supports the theory of adult neurogenesis in the SVZ (Eriksson et al., 1998; Quiñones-Hinojosa et al., 2006; Jurkowski et al., 2020).

In addition to its emerging role as a source of adult neurogenesis, the SVZ is essential for the development of the cortex during embryonic neurogenesis. Together with the ventricular zone (VZ), the SVZ is the main source of newborn neurons in embryogenesis. Disruptions to this process that result in dysfunctional migration or over proliferation of neuronal progenitors have been linked to ASD-like symptoms (Purves, 2001; Fang et al., 2014; Herzine et al., 2016; Yao et al., 2016; Guarnieri et al., 2018). Essential in ASD development is the process of radial migration, which is mediated by radial glial cells (RGCs) in the SVZ, allowing newly differentiated neurons to migrate from the SVZ through the established layers of the cortex to their final destination on the pial wall (Purves, 2001; Wu et al., 2014; Guarnieri et al., 2018; Casingal et al., 2022). Alterations of the RGC scaffold and signaling molecules through genetic/epigenetic and/or environmental changes results in structural malformation, disrupted cell-cell signaling, and malfunction of cortical circuitry, resulting in an apparently macroscopically normal cortex that nonetheless has aberrant neuronal wiring (Wu et al., 2014; Yao et al., 2016; Guarnieri et al., 2018; Ciarrusta et al., 2020; Casingal et al., 2022). Indeed, in our recent study, we identified robust differences in the DNA methylation levels of genes preferentially involved in neuronal differentiation, axon specification, and migration, in the SVZ of idiopathic ASD cases, that in some instances remarkably resembled the methylation states at earlier time points in fetal brain development (Corley et al., 2019). Altogether, these findings suggest an early developmental failure to establish appropriate epigenomic landscapes requisite for transcriptional programing of neurogenesis and development, likely impacting the neuronal stem cell (NSC) compartment in the SVZ.

A related structure in the brain that has also been connected to ASD is the insular cortex (Caria and de Falco, 2015; Jd et al., 2018; Nomi et al., 2019). Most neurons in the insular cortex are derived from the SVZ during development, particularly by the pallial-subpallial boundary, and atypical patterns of activation of the insula have been observed in ASD (González-Arnay et al., 2017). It has long been observed that ASD is associated with abnormal interconnectivity of the CNS, with hyper frontal lobe local connectivity and decreased long distance connectivity to other parts of the brain (Courchesne and Pierce, 2005; Ciarrusta et al., 2020). A meta-analysis of functional magnetic resonance imaging (fMRI) studies determined that ASD is associated with hypoactivation of the right anterior insula during social processes (Di Martino et al., 2009). The insular cortex has been related to cognitive functions including consciousness and self-awareness, language processing, and interpersonal experiences (Mufson and Mesulam, 1984; Craig A. D., 2002); it also appears to play important roles in emotional states and primary interoceptive activity (Craig A. D. B., 2010; Gasquoine, 2014). MRI studies have reported abnormalities in the insular cortex in patients with ASD (Patriquin et al., 2016; Parellada et al., 2017), and recently also reported that MRI-based measures related to tissue microstructures in the insula are linked to more global connectional abnormalities in ASD (Menon et al., 2020).

Anatomical and morphological changes observed in the brains of ASD adults and children supports the classification of ASD as a neurodevelopmental disorder and correlates with severity of symptoms. This atypical neural connectivity has been supported by postmortem brain analysis that showed neurodevelopmental abnormalities including altered neurogenesis and defective neuronal migration reflected by the disorganization and abnormal cortical laminar cytoarchitecture (Wegiel et al., 2010; Ecker et al., 2012; Stoner et al., 2014). MEG, EEG, and MRI studies have demonstrated differences in long range and local circuitry of ASD brains, with higher levels of local connectivity and lower long range connectivity (O’Reilly et al., 2017; Ciarrusta et al., 2020). General changes include a decrease in neuronal size and volume, abnormal migration and maturation of neurons, altered density of dendritic spines, and the appearance of swollen axon terminals, as well as macrocephaly and changes to cortical surface area (Ohta et al., 2016; Varghese et al., 2017; Shiohama et al., 2022). ASD brains are also found to have signs of neurodegeneration, neuronal loss, and inflammation, with thinning of the superior parietal, temporal, and frontal cerebral cortexes in teenagers with ASD (Kotagiri et al., 2014). One of the more consistent areas observed to have changes in brains of patients with ASD is the SVZ and its cytoarchitecture. While the overall structure of the SVZ has not been found to be significantly different, density and thickness of the hypocellular gap has been observed to be decreased in ASD (Pearson et al., 2013; Kotagiri et al., 2014).

Recently, studies in pluripotent stem cells (iPSC) derived from individuals with an ASD related disorder like Rett syndrome (RTT) or Fragile X syndrome (FXS) (Hodges et al., 2020) showed defects in neuronal maturation and aberrant neuronal differentiation (Kim et al., 2011; Sheridan et al., 2011, p. 1). Additionally, this approach has been used to analyze ASD-associated genes which lose their function, such as SHANK2 or SHANK3, revealing impairment in neuronal morphology and transcriptomic changes in neurodevelopment-associated pathways (Huang et al., 2019; Zaslavsky et al., 2019). Similarly, neurodevelopmental alterations are present in iPSC derived from idiopathic ASD subjects, where morphometric analyses revealed a developmental acceleration in differentiating neurons and the transcriptomic signature exhibited a temporal dysregulation in a group of genes involved in biological processes such as neuron differentiation, cell morphogenesis and synaptic signaling (Schafer et al., 2019). Patterns of neuronal migration observed using MRI demonstrates a persistence of radial coherent structures in a post-natal brain that mirrors those observed in embryonic weeks 15–28 (Kamiya et al., 2014), which implies disruption of radial scaffold or unit formation and function of radial glial progenitors in a developing brain with a mutation in TUBA1A, a protein associated with microcephaly, lissencephaly, intractable epilepsy, and developmental delay (Casingal et al., 2022). NSCs within the SVZ display more proliferative markers, including Ki-67 and BrdU, but appear to stall before completing neurogenesis or mitosis (Marchetto et al., 2017; Varghese et al., 2017; Guarnieri et al., 2018; Grasselli et al., 2020).

Structural MRI studies have shown subtle developmental disturbances of gyral folding that can be linked to autism (Levitt et al., 2003), and subtle abnormal white matter (WM) development, such as abnormal asymmetry (Chiron et al., 1995) suggests altered brain connectivity in various developmental disorders including ASD (Owen et al., 2013; Roine et al., 2013; Abdel Razek et al., 2014; Levman and Takahashi, 2015). However, the whole picture of ASD brain development is still elusive. In addition, diffusion MRI (dMRI) tractography can be used to detect three-dimensional pathways based on microstructural, regional water diffusivity in the entire brain mantle. In traditional studies, dMRI tractography has been used to observe WM pathways. However, dMRI has recently also revealed important developmental processes in humans such as radial glial pathways in the developing cortex (Takahashi et al., 2012; Kolasinski et al., 2013; Xu et al., 2014; Miyazaki et al., 2016; Vasung et al., 2017; Das and Takahashi, 2018). Given that postnatal neuronal migration has been observed as neuronal chains tightly wrapped by astrocytes along blood vessels (Kaneko et al., 2011), it is likely that dMRI tractography can also detect postnatal neuronal migration streams in the brain.

Given the role of the SVZ in cortical development and our prior observations, we hypothesized that ASD in childhood may originate from epigenetic alterations in the NSC compartment of the SVZ, resulting in altered neuronal trajectory in differentiation, migration, and maturation observed in the cortex that could be imaged with structural and dMRI.

## Materials and methods

### *Ex vivo* MRI

#### Specimens

The research was conducted in accordance with the Helsinki Declaration and approved by the Boston Children’s Hospital Human Research Committee. Brain specimens of two children with ASD and two children with no neurological/pathological findings were received from the University of Maryland Brain and Tissue Bank, under a material transfer agreement between the Brain Bank and the Boston Children’s Hospital.

Case #1, 5308, is a 4 years and 183 days old white male, diagnosed with autism, whose cause of death is skull fractures due to blunt force injuries by being struck by a car. His final neuropathological diagnosis included: (1) blunt force head injuries, (a) multifocal subarachnoid hemorrhage, and (b) intermediate contusions, cerebral WM tracts, and brainstem, (2) Possible microdysgenesis, temporal lobe. His Autism Diagnostic Interview-Revised (ADI-R) results were, Section A = 17, Section B verbal = 14, Section B non-verbal = 14, Section C = 4 and Section D = 3. The postmortem interval was 21 h.

Case 2, #1349, is a 5 years and 222 days old white male, diagnosed with autism, whose cause of death was drowning. The gross appearance of his brain did not indicate edema or discoloration and appeared normal. His final diagnosis was a well-developed brain with no significant neuropathological changes. The postmortem interval was 39 h.

Case 3, #M4021M, is the tissue of a 3 years and 114 days old African American male, diagnosed with autism, who died from a drowning accident. The family history report included a mentally ill maternal cousin, a brother with a speech delay, and a father that stutters. The neurology report stated a grossly normal brain and the autopsy reported pulmonary edema and congestion, as well as cerebral edema. The postmortem interval was 15 h.

Case 4, #M4029M, is the tissue of a 3 years and 274 days old African American male with autism and delayed speech, who died from a drowning incident. Neurology report included anoxic-ischemic encephalopathy in the basal ganglia and acute brain edema. The autopsy found pulmonary edema and congestion, as well as cerebral edema. The postmortem interval was 13 h.

Case 5, #6032, is a control for Case 1 and Case 2, a 4 years and 51 days old white male, whose cause of death was head and neck injuries by an accident. The report included subarachnoid hemorrhage, right lateral frontal lobe, and subarachnoid hemorrhage, ambient cistern. There was no gross and pathological finding during autopsy. The postmortem interval was 25 h.

Case 6, #5282, is the control for Case 3, and is the brain tissue of a 2 years and 305 days old Hispanic male. Prior to death from choking, the child was reported healthy and was not on any medications. Cause of death (choking) would have no detrimental effect on the WM pathways within the brain nor the gray matter (GM) examined in this study. No previous neurological conditions were found; therefore, the brain is an appropriate subject for a control in this case. The postmortem interval was 16 h.

Case 7, #5608, the control for Case 4, is a 3 years and 194 days old Caucasian male who was born with a ventricular septal defect. Prior to death, the child had three open heart operations and intestinal surgery. Neurology record reported a small brain for his age, but otherwise grossly normal. Cause of death (drowning) would have no detrimental effect on the WM pathways within the brain nor the GM examined in this study. No previous neurological conditions were found; therefore, the brain is an appropriate subject for a control in this case. The postmortem interval was 29 h.

The brain slabs were all from the right hemisphere, and their thicknesses were approximately 1.5 cm. Cases 3, 4, 6, and 7 were previously used in our paper (Wilkinson et al., 2016), but newly analyzed with epigenetic procedures.

#### Preparation for MRI scan

The slabs were prepared for the MRI scans by being placed into individual Ziploc bags with Fomblin oil, and then placed parallel to each other in a box with plastic boards placed in between the slices. The scans were done at the Athinoula A. Martinos Center for Biomedical Imaging.

#### MRI scan parameters

Diffusion-weighted data were acquired over two averages using a steady-state free-precession (SSFP)-based diffusion sequence (trufi) (TR/TE = 24.82/18.76 msec, α = 60°, bandwidth = 100 Hz/px) (McNab and Miller, 2008), using a 3 Tesla Siemens TIM Trio MRI machine with a 32-channel head coil. Imaging matrix was 200 × 400 × 200 for the slice specimens, and 176 × 128 × 192 for the whole brain. Slab thickness 192 mm with 240 slices (800 um thick slices), 200 × 200 matrix with 160 mm square Field of View (FOV) (800 μm × 800 μm in-plane resolution) for whole brain. Diffusion weighting was performed along 44 directions (*b* = approximately 2,000 s/mm^2^) with 4 *b* = 0 images. The diffusion directions were generated by electrostatic repulsion on the surface of a sphere to ensure approximately equidistant spacing. Total scan time was 17 h 35 min 24 s. With an SSFP diffusion scan, achieving a much higher *b*-value is difficult, given the long scans and constraints on gradient heating. The use of SSFP was necessary to achieve high-resolution *ex vivo* diffusion and is relatively free of distortions.

#### Reconstruction and identification of tractography pathways

Pathways in GM and WM were reconstructed using Diffusion Toolkit and visualized in TrackVis software.^1^ A streamline algorithm for diffusion tractography was used (Xue et al., 1999), as in previous publications (Takahashi et al., 2010, 2012). The term “streamline” refers to connecting tractography pathways using a local maximum (of tensors in DTI) or maxima [of orientation distribution function (ODF) in HARDI]. This method is valid for both DTI and HARDI. We used the local ODF maxima to produce HARDI tractography pathways. Trajectories were propagated by consistently pursuing the orientation vector of least curvature. Tracking was terminated when the angle between 2 consecutive orientation vectors was greater than the given threshold (60°) or when the fibers extended outside the brain surface. For the latter determination, brain mask images created by Diffusion Toolkit were used in order to determine coherence within the brain and not in the surrounding immersion fluid. Brain mask volumes were used to terminate tractography structures instead of the FA threshold because progressive myelination and crossing fibers in the developing brain can result in low FA values that may potentially incorrectly terminate tractography tracing in the GM (Wedeen et al., 2005; Takahashi et al., 2010, 2012). Although we have been using this strategy in our past studies, the use of an FA threshold when performing diffusion tractography is still a common tractography method, so our approach is unique in that sense. Since there are relatively large low FA regions in immature WM, it is important to consider not using an FA threshold for tractography in infants and children. This issue may also apply to tractography in adults as FA values tend to decrease potentially to lower values than a given threshold toward the edge of WM.

**FIGURE 1:**
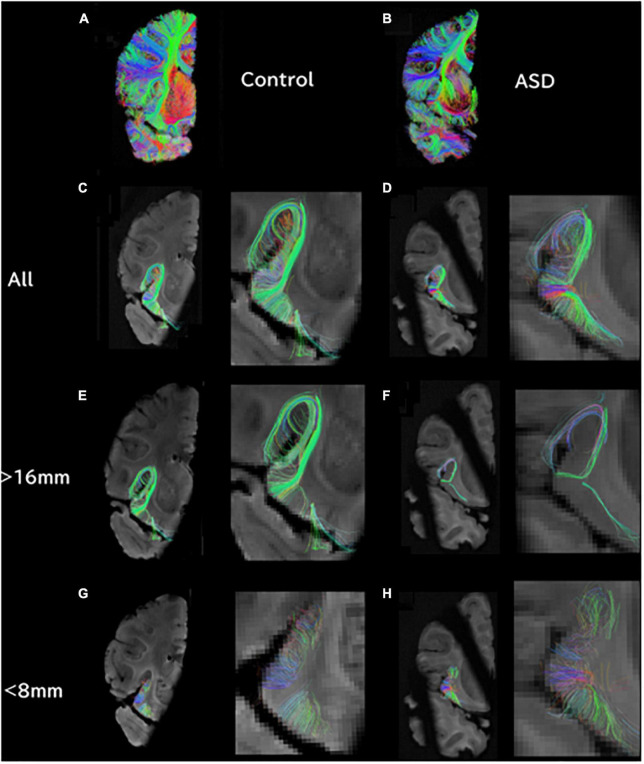
At the level of the insula/thalamus, longer tractography pathways are decreased and shorter pathways are increased in ASD. **(A)** Overall tractography pathways in right hemisphere of Control (#6032; 4 years and 51 days old). **(B)** Overall tractography pathways in right hemisphere of a brain diagnosed with ASD (#5308; 4 years and 183 days old). **(C)** Tractography pathways from/to the insula at level of insula/thalamus in control. **(D)** Tractography pathways from/to the insula at level of insula/thalamus in ASD. **(E)** Long tractography pathways (>16 mm) from/to the insula of control. **(F)** Long tractography pathways (>16 mm) from/to the insula of ASD. **(G)** Short tractography pathways (<8 mm) within the insula/thalamus of control. **(H)** Short tractography pathways (<8 mm) within the insula/thalamus of ASD.

**FIGURE 2:**
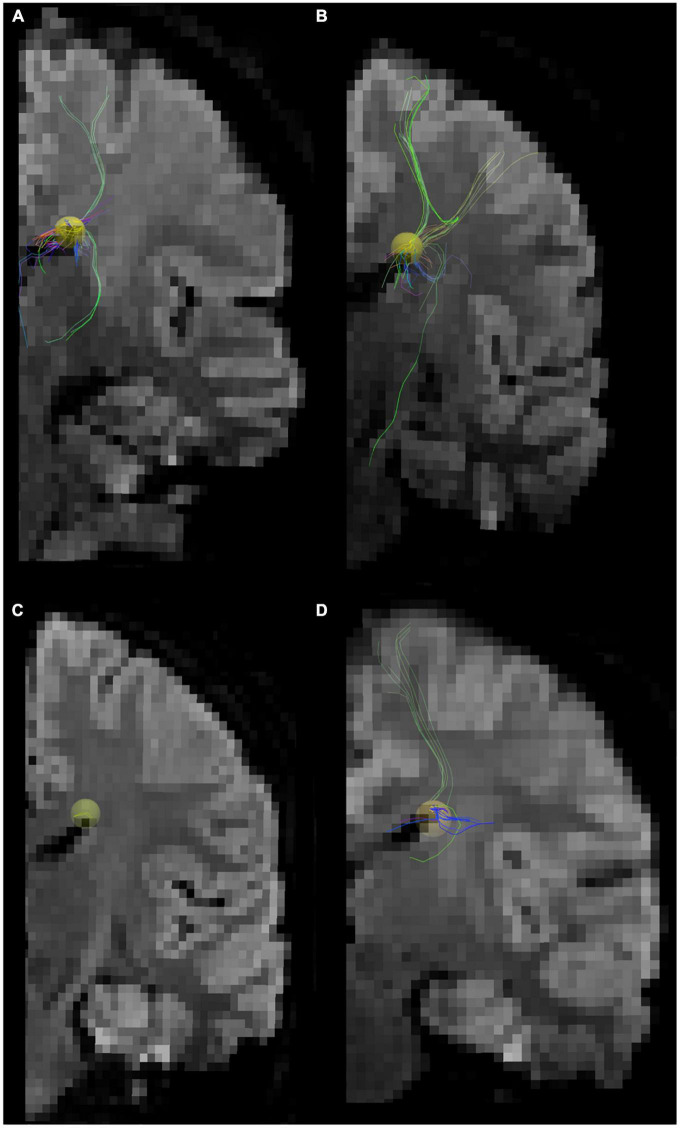
Tractography pathways associated with the dorsal SVZ are more sparse and fewer in ASD compared to control *in vivo*. Tractography pathways associated with the dorsal SVZ in **(A,B)** 16YO male controls and **(C,D)** 16YO male patients diagnosed with ASD. We examined *in vivo* diffusion tractography in coronal slabs at the level of the insula and thalamus. Yellow spheres (4 mm radius) in each brain image were created in the dorsal SVZ as regions of interest (ROIs) to detect pathways from/to the ROIs.

**FIGURE 3:**
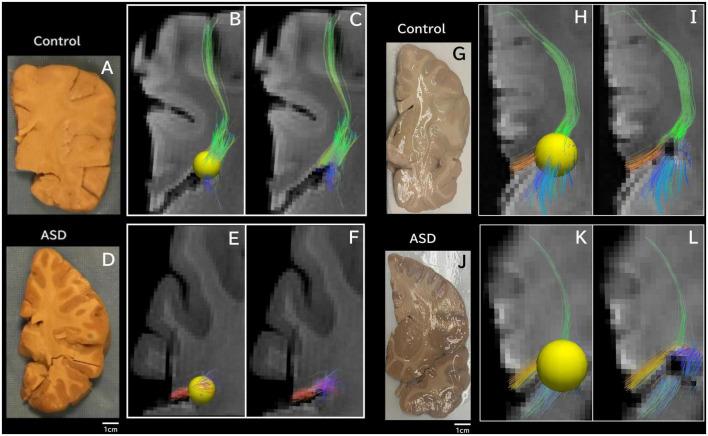
Tractography pathways associated with the dorsal SVZ are more sparse and fewer in ASD compared to control *ex vivo*. Tractography pathways associated with the dorsal SVZ in **(A–C)** control 6032; 4 years and 51 days old; **(D–F)** ASD #M4021M; 3 years 114 days old; **(G–I)** Control #5282; 2 years 308 days old; and **(J–L)** ASD #5308; 4 years 183 days old. Yellow spheres (4 mm radius) in each brain image were created in the dorsal SVZ as regions of interest (ROIs) to detect pathways from/to the ROIs. Tractography pathways without the ROIs are shown in **(C,F,I,L)**. Brain images **(A,D)** were previously published (Wilkinson et al., 2016).

**FIGURE 4:**
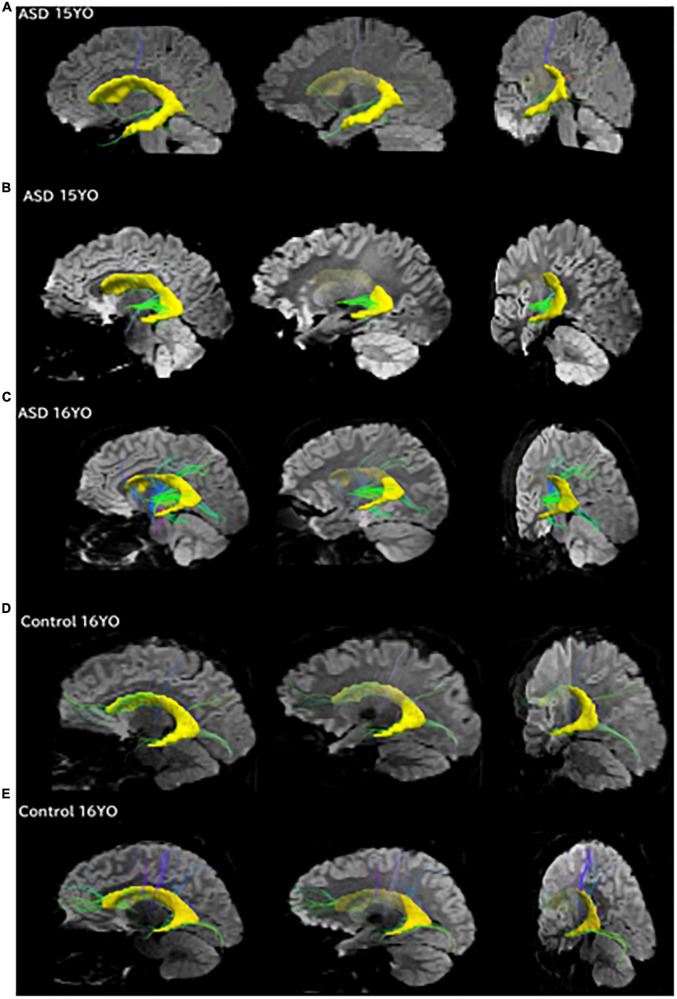
Pathways between the posterior SVZ and the insula were absent or fewer in controls compared to ASD patients. Tractography pathways from the entire SVZ (yellow) in patients with ASD and control at three different slices. **(A)** 15-year-old with ASD; **(B)** 15-year-old with ASD; **(C)** 16-year-old with ASD; **(D)** 16-year-old control; **(E)** 16-year-old control. Images from each patient are shown at mid-sagittal slices (left column), lateral sagittal slices at the level of the insula (middle column), and posterior oblique views with mid-sagittal slices and coronal slices at the level of the insula (right column) are shown. Pathways between the posterior SVZ and the insula were absent or fewer in controls compared to ASD patients.

The color-coding of tractography pathways was based on a standard red-green-blue (RGB) code that was applied to the vector in each brain area to show the spatial locations of terminal regions of each pathway. For visualization purpose, multiple ways of the color-coding were used (red for right-left, blue for dorsal-ventral, and green for anterior-posterior in Figures 1–3, and red for right-left, green for dorsal-ventral, and blue for anterior-posterior in Figure 4), in order to clearly visualize the pathways from the posterior SVZ to the insula in Figure 4.

We showed all the detected tractography pathways in Figures 1–4, using only insular ROIs (Figure 1), spheres in the dorsal SVZ region (Figures 2, 3), and entire SVZ ROIs (Figure 4). For Figure 2, tractography pathways that do not directly come from/go to the edge of the ventricle were excluded using additional spherical ROIs. No length threshold was used.

### Clinical MRI

In this study, we analyzed a part of the unpublished data from our previous work (Levman et al., 2019).

#### Participants

Following approval by BCH’s Institutional Review Board (informed consent was waived due to the lack of risk to participants included in this retrospective analysis), the clinical imaging electronic database at BCH was reviewed for the present analysis from 01/01/2008 until 02/24/2016, and all brain MRI examinations of participants aged 0–32 years at the time of imaging were included for further analysis if autism was indicated in the participant’s electronic medical records. More detailed diagnostic information (such as ADI-R and ADOS gold standard diagnoses) were not available in this dataset and this issue is addressed in more detail in the limitations section of the Discussion. Examinations deemed to be of low quality (because of excessive participant motion, large metal artifact from a participant’s dental hardware, lack of a T1 structural imaging volume providing diagnostically useful axial, sagittal and coronal oriented images, etc.) were excluded from the study. Examinations that were inaccessible for technical reasons were also excluded. This yielded 1,003 examinations from 781 autistic participants. Typically developing participants were assembled retrospectively in a previous analysis (Levman et al., 2017) by selecting participants on the basis of a normal MRI examination, as assessed by a BCH neuroradiologist, and whose medical records provided no indication of any neurological problems (participants with any known disorder were excluded such as autism, cerebral palsy, traumatic brain injury, cancer, developmental delay, multiple sclerosis, tuberous sclerosis complex, stroke, neurofibromatosis, cortical dysplasia, epilepsy, attention deficit hyperactivity disorder, etc.). Participants with any form of cancer were also excluded to avoid data exhibiting growth trajectories negatively affected by treatments such as chemotherapy. The same exclusion criteria applied to the autistic population were also applied to the typically developing participants. This yielded 993 examinations from 988 typically developing participants. Table 1 provides a breakdown of the autistic and healthy populations divided by age groups used in the statistical analysis under the Methods section.

**TABLE 1:** Demographic breakdown of our two populations.

	Age (years)				
	0 to 5	5 to 10	10 to 15	15 to 20	20 +
Neurotypical	*M* = 71, *F* = 68, 2.59 ± 1.43 years	*M* = 124, *F* = 137, 7.63 ± 1.41 years	*M* = 115, *F* = 177, 12.41 ± 1.41 years	*M* = 80, *F* = 194, 16.70 ± 1.11 years	*M* = 4, *F* = 23, 22.21 ± 2.63 years
Autistic	*M* = 283, *F* = 96, 2.89 ± 1.89 years	*M* = 237, *F* = 79, 7.23 ± 1.47 years	*M* = 150, *F* = 30, 12.17 ± 1.44 years	*M* = 92, *F* = 19, 16.97 ± 1.42 years	*M* = 10, *F* = 6, 21.27 ± 1.01 years

Count of males (M) and females (F), each are followed by the average and standard deviation of this group’s ages in years (years). This table was adapted from our previous work (Levman et al., 2019).

#### MRI data acquisition and pre-processing

Participants were imaged with clinical 3 Tesla MRI scanners (Skyra, Siemens Medical Systems, Erlangen, Germany) at BCH yielding T1 structural volumetric images accessed through the Children’s Research and Integration System (Pienaar et al., 2017). Because of the clinical and retrospective nature of this study, there is variability in the pulse sequences employed to acquire these volumetric T1 examinations. Spatial resolution varied in the x and y directions from 0.219 to 1.354 mm (mean: 0.917 mm, standard deviation: 0.124 mm). Through-plane slice thickness varied from 0.500 to 2.000 mm (mean: 0.996 mm, standard deviation: 0.197 mm). Strengths and limitations of the large-scale varying MR protocol approach taken in this study are addressed in the Discussion. Motion correction was not performed, but examinations with substantial motion artifacts were carefully excluded based on visual assessment. These motion corruption exclusions were performed to compensate for the additional difficulties autistic patients have in remaining still during image acquisition relative to the typically developing population. T1 structural examinations were processed with FreeSurfer (Fischl, 2012) using the recon_all command which aligns the input examination to all available atlases. Those atlases that include cortical thickness (CT) measurements were included for further analysis (aparc, aparc.a2009, aparc.DKTatlas40, BA, BA.thresh, entorhinal_exvivo). These combined atlases include definitions of 331 cortical regions in the brain. Each FreeSurfer output T1 structural examination was displayed with label map overlays and visually inspected for quality of regional segmentation results. If FreeSurfer results were observed to substantially fail, they were excluded from this analysis [i.e., FreeSurfer regions-of-interest (ROIs) that do not align to the MRI and examinations where major problems were observed with an ROI such as a cerebellar segmentation extending far beyond the extent of the cerebellum].

#### Statistical analysis

This study included the acquisition of 662 regionally distributed CT measurements per imaging examination, as extracted by FreeSurfer’s recon-all command which processes the input examination with all available atlases (Fischl, 2012). This included extracting measurements of both average and the standard deviation of within-region CT for each supported GM region. This includes all sub-regions of the brain supporting CT measurements across all FreeSurfer supported atlases. Study participants were divided into four groups based on age: early childhood (0–5 years old), late childhood (5–10 years old), early adolescence (10–15 years old), and late adolescence (15–20 years old). We had very few participants greater than 20 years old and so did not include them in a separate group, however, all scatter plots included all participants regardless of age to facilitate visual comparison. We are interested in the diagnostic potential of these clinically acquired measurements and so each measurement (as extracted by FreeSurfer) within each age range was compared in a group-wise manner (autism compared with neurotypical) with receiver operating characteristic (ROC) curve analysis which is summarized with the area under the ROC curve (AUC) (Youngstrom, 2014), Cohen’s d statistic (positive/negative values indicate a higher/lower average value in the autistic population relative to the neurotypical population) and a *p*-value based on the standard t-test for two groups of samples. The *p*-value was selected as an established method to demonstrate that it is unlikely that our findings were the result of random chance, Cohen’s D was selected as it is the most established method to assess effect sizes and the AUC was selected to extend our analysis to the assessment of diagnostic potential. This yielded a total of *m* = 2,648 group-wise comparisons, yielding a Bonferroni corrected threshold for achieving statistical significance of p < 0.05/*m* = 1.89e^–5^.

In order to confirm that the findings reported are the result of group-wise differences between the autistic and neurotypical participants, a statistical model was constructed based on multivariate regression (using the my regress function in MATLAB), adjusting each measurement within each age range in order to control for group-wise differences in age, gender, estimated total intracranial volume and the leading comorbid status of the most common secondary conditions from our two groups: headaches (7% in the autistic group, 19% in the neurotypical group), ADHD (16% in the autistic group, 0% in the neurotypical group), epilepsy (13% in the autistic group, 0% in the neurotypical group), global developmental delay (26% in the autistic group, 0% in the neurotypical group), migraines (3% in the autistic group, 23% in the neurotypical group), and abdominal pain (14% in the autistic group, 11% in the neurotypical group). This model was used to adjust each CT (mean and standard deviation) measurement, in order to evaluate whether group-wise differences between our autistic and typically developing populations are the result of age, gender, intracranial volume or comorbid effects.

Our dataset includes very few exams of participants older than 20 years of age and so this range was not included in the group-wise age-dependent statistical analyses because of the small sample size but was included in all scatter plots for reference. Age-dependent ROC analysis allows us to assess the diagnostic potential of any given FreeSurfer measurement and Cohen’s d statistic provides useful effect size measurements at multiple developmental stages. Scatter plots were created to visually present measurements-of-interest from male and female participants as they vary with age. Trend lines in all scatter plots were established with a rolling average (*K* = 150) implemented in MATLAB (Natick, MA, USA).

### Sequencing

#### Postmortem brain tissue samples

Samples of the right insular cortex were as described above. Two age matched frozen postmortem brain tissue specimens, AN13287 and UMB1135, were obtained from National Institute of Child Health Human Development Brain and Tissue Bank for Developmental Disorders (University of Maryland) and Harvard Brain Tissue Resource Center (Boston, MA, USA) through the Autism Tissue Program of Autism Speaks. More detailed information regarding these specimens can be found in our previous work (Corley et al., 2019). To control for anatomical variability, landmarks were chosen and marked with India ink during dissections at each brain bank. Tissue blocks (2.0 cm^3^) were excised from a brain region containing the SVZ region of the lateral ventricles and contained portions of the head of the caudate and corpus callosum from fresh frozen coronal sections. A 5-mm tissue punch containing the SVZ region per sample was used for DNA and RNA extractions. Specimens were de-identified and the study was reviewed by the University of Hawaii (UH) Human Studies Program under application CHS#2016-30171. They determined that this study did not qualify as human subjects research, and thus did not require review and approval by the Human Studies Program or a UH Institutional Review Board (IRB).

#### Whole genome bisulfite sequencing

DNA was isolated simultaneously with RNA from postmortem SVZ brain samples using AllPrep DNA/RNA/miRNA Universal Kit (80224, QIAGEN) as described above. DNA samples were diluted using EB buffer from the kit to 15 ng/mL and sent to Macrogen, Inc (Seoul, Republic of Korea) for paired-end whole genome Bisulfite sequencing (WGBS). Macrogen performed a quality control check of all samples before preparing libraries by random fragmentation of the DNA and 5′ and 3′ adapter ligation. Adapter-ligated fragments were PCR amplified and gel purified. Sequencing was performed using the Illumina NovaSeq platform.

### Expression analysis

#### Nuclei extraction and immunostaining

Postmortem SVZ human brain tissue samples were thawed on ice and transferred into a 1.5 mL Eppendorf tube. Tissue was homogenized in nuclear extraction buffer (0.32 M Sucrose, 10 mM Tris–HCl pH8.0, 5 mM CaCl_2_, 3 mM MgCl_2_, 1 mM DTT, 0.1 mM EDTA and 0.1% Triton X-100) using a disposable pellet pestle (∼30 strokes).

Nuclei were isolated by using a discontinuous sucrose gradient. The homogenized tissue solution was transferred into a 50 mL centrifuge tube (3115-0050, Thermo Fisher Scientific) and a sucrose solution (1.8 M sucrose, 10 mM Tris–HCl pH8.0, 3 mM MgCl_2_ and 1 mM DTT) was added at the bottom to form the gradient. Samples were centrifuged at 75600 × *g* at 10°C, for 1 h in a Beckman Coulter centrifuge Avanti J-20 XPI with a fixed angle JA-25.50 rotor. Nuclei were stained with a mouse anti-neuronal nuclei (NeuN) Alexa Fluor 488 conjugated monoclonal antibody (Alexa488NeuN antibody) (MAB377X, Millipore) and DAPI (D1306, Thermo Fisher Scientific).

#### FACS

Alexa488NeuN+, DAPI+, and Alexa488NeuNDAPI+ nuclei were sorted using a BD FACSAria III flow cytometer. Parameters and data analysis were established using BD FACSDiva software version 8.0.1 (BD Biosciences, San Jose, CA, USA). Alexa488NeuN antibody fluorescence was detected with the 488 nm laser and a 530/30 filter and DAPI fluorescence was detected using the 407 nm laser and a 450/40 filter. Nuclei were sorted using a 100 μm nozzle at 20 psi with a drop-drive frequency of 29.2 kHz and collected in 1.5 mL Eppendorf tubes with DPBS buffer (14287-080, Gibco).

#### qPCR assays

Postmortem SVZ human brain tissue samples and sorted nuclei were used to extract DNA and RNA using the AllPrep DNA/RNA/miRNA Universal kit (80224, QIAGEN). RNA was treated with DNase and transformed to cDNA using SuperScript™ IV Vilo Master Mix Kit (11756050, Thermo Fisher Scientific, Vilnius, Lithuania) according to the protocol of the manufacturer. qPCR assays were performed in a StepOnePlus Real-Time PCR System using 40 ng of cDNA from each sample and TaqMan Fast Advanced Master Mix (4444557, Thermo Fisher Scientific) according to the manufacturer’s instructions, using a passive reference of ROX, and analyzed using StepOne Software v2.3 (Thermo Fisher Scientific). TaqMan assays were selected from existing assays available from Thermo Fisher Scientific specific to human with probes spanning exons. Probes labeled as best coverage were preferred. During qPCR, samples were incubated for 2 min at 50°C, then the polymerase was activated at 95°C for 2 min. A total of 40 cycles were performed of 1 s at 95°C and 20 s at 60°C. Once complete, samples were held at 4°C. Cò results were normalized to Actinβ (ActB) amplification, performed in the same assay, and the estimated percent expression was calculated relative to ActB using the delta-Ct method. The TaqMan Assays (Thermo Fisher Scientific) used for this study included: GFAP (Hs00909233_m1), SLC1A3 (Hs00904823_g1), SOX2 (Hs01053049_s1), NES (Hs04187831_g1), ASCL1 (Hs00269932_m1), DCX (Hs00167057_m1), RBFOX3/NeuN (Hs01370653_m1), LIFR (Hs01123581_m1), RBP1 (Hs01011512_g1), FAM107A (Hs00200376_m1), EP300 (Hs00914223_m1), SEPP1 (Hs01032845_m1), DMNT1 (Hs00945875_m1), DMNT3a (Hs00602456_m1), DMNT3b (Hs00171876_m1), and ACTB (Hs01060665_g1).

### Sequencing analysis

#### Whole genome bisulfite sequencing analysis

The WGBS reads were first processed using nf-core/methylseq bioinformatics analysis pipeline to extract the DNA methylation levels for all the samples (Ewels et al., 2020). In this pipeline, the adaptors and reads with low-quality regions were trimmed or removed by Trim Galore! and the quality of the reads were monitored by FASTQC before and after the trimming. Bisulfite alignment of the reads to hg38 assembly of human reference genome and methylation calling were performed using Bismark (Krueger and Andrews, 2011). The Bismark coverage reports, containing actual read coverage of detected methylated or unmethylated reads at each position, were used as the inputs for the next step analysis performed by R package RnBeads (Müller et al., 2019). The methylation sites with coverage less than 5 reads or with high coverage marked as outliers were removed, while the sites with more than 3 overlapped SNPs or too many missing values were filtered out as well. The rest sites were annotated based genomic regions including CpG sites, tilling regions, genes, and promoters. The differentially methylated region (DMR) analysis can be performed in each level of these region types. Differential methylation on one of the region types was computed based on three metrics: the mean difference in means across all sites in a region of the two groups (unaffected, typically developing “control” vs. ASD) being compared, the mean of quotients in mean methylation, and a combined *p*-value calculated from all site *p*-values in the region. A combined rank is computed as the maximum (i.e., worst) value among the three ranks, which were assigned to each region based on the three metrics used. The smaller the combined rank for a region, the more evidence for differential methylation it exhibits. DMRs (genes, promoters) were selected according to the combined rank of a given region and GO enrichment analysis was performed based on these DMRs to predict the functions that might be affected under ASD. Of all sites that were quantified for methylation, we sought to identify sites with significant DNA methylation differences (δ of the β value) between the ASD and control groups (*P* < 0.05) using the resampling-based empirical Bayes Methods permutation approach with *P* < 0.05 and filtered for sites with absolute average methylation differences greater than 10% (δ) between the ASD and control groups. This approach reduces the false discovery rates for non-normally distributed array-based data and offers higher statistical power. Incorporating differences at a threshold of 10% absolute difference in DNA methylation also diminishes the likelihood of random technical errors from true biological differences, increasing the confidence in detecting differences in DNA methylation (Dedeurwaerder et al., 2014). We consider these CpGs as differentially methylated loci (DML).

#### IPA analysis

To predict biological functions and gene regulatory networks, differentially methylated or expressed sites identified through WGBS were submitted to the Ingenuity Pathway Analysis software [IPA; QIAGEN; (Krämer et al., 2014]. Gene regulatory networks with a log_2_ fold change of DNAm level were highlighted.

## Results

### Clinical and *ex vivo* MRI—Insular cortex

**FIGURE 5:**
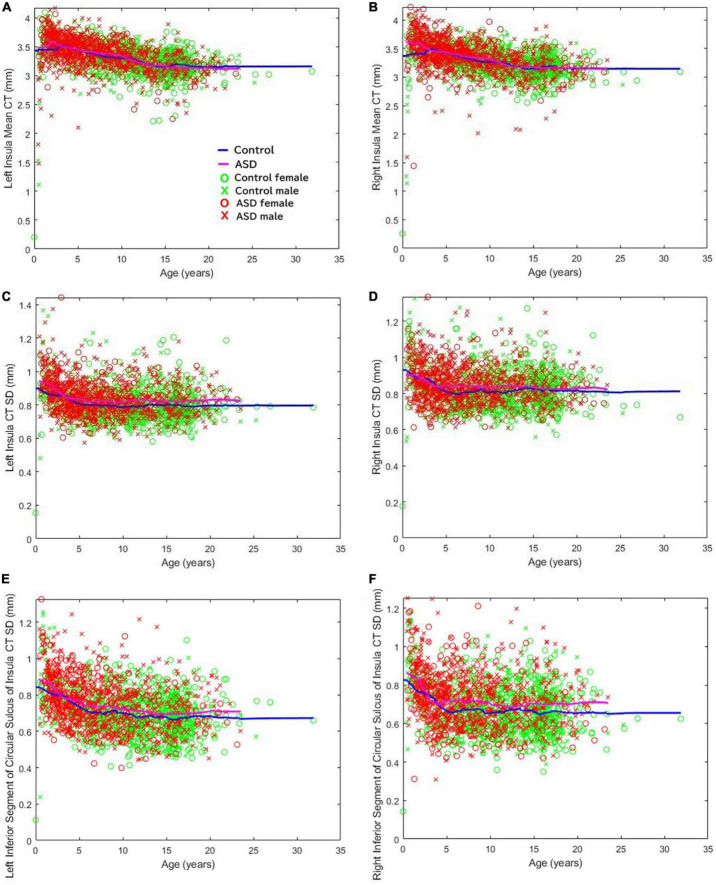
Cortical thickness variability of several regions of the insula were found to be elevated in ASD. The mean cortical thickness of the **(A)** left and **(B)** right insula. Standard deviation of the cortical thicknesses of the **(C)** left and **(D)** right insula. Standard deviation of the thicknesses of the **(E)** left and **(F)** right inferior segment of the circular sulcus of the insula across all ages available (Levman et al., 2019).

Previous clinical imaging analyses conducted at Boston Children’s Hospital, focusing on both distributed volumetric analysis of an autistic population (Levman et al., 2018) as well as a detailed CT analysis (Levman et al., 2019), supports the assessment of insular abnormalities in newborn and pediatric populations. We did not specifically report about the insular cortex in those studies. Figure 5 illustrates the mean CT of the left (a) and right (b) insula, the standard deviation of the CT of the left (c) and right (d) insula, and the standard deviation of the thicknesses of the left (e) and right (f) inferior segment of the circular sulcus of the insula across all ages available (Levman et al., 2019). Note that CT variability, assessed by the standard deviation, represents a within-region within-patient regionally specific biomarker of cortical structure (Levman et al., 2019). The thickness variability of several regions of the insula were found to be elevated in the autistic group: the left inferior segment of the circular sulcus of the insula (ages 5–10, *d* = 0.27, *p* < 0.002; ages 10–15, *d* = 0.22, *p* < 0.02); the right inferior segment of the circular sulcus of the insula (ages 5–10, *d* = 0.39, *p* < 0.000003; ages 10–15, *d* = 0.19, *p* < 0.04; ages 15–20, *d* = 0.38, *p* < 0.0007); the left cortical insula (ages 5–10, *d* = 0.32, *p* < 0.0002; ages 10–15, *d* = 0.32, *p* < 0.0007); and the right cortical insula (ages 5–10, *d* = 0.36, *p* < 0.00002; ages 10–15, *d* = 0.31, *p* < 0.002). Although we observed extensive insular irregularities throughout the 5–15 year age groups, it should be noted that the previous analyses (Levman et al., 2018, 2019) were performed prior to the availability of modern infant FreeSurfer atlases (Zöllei et al., 2020), and so a more fine-tuned analytic approach validated on this youngest age group may result in the discovery of insular irregularities in these early age groupings as well. Findings imply that insular irregularities extend into childhood and may imply that the findings outlined in this manuscript could be associated with known emotional regulation symptoms in autism.

*Ex vivo* diffusion tractography also showed an overall decrease in the number of pathways in coronal slabs at the level of the insula and thalamus (Figures 1A, B). Tractography pathways from/to the insular cortex were identified in the control (Figures 1C, E, G) and in the patient with ASD (Figures 1D, F, H). The ASD specimen also showed relatively sparse pathways (Figure 1D) compared to the control (Figure 1C). Long tractography pathways (>16 mm) were found both in the control (Figure 1E) and in ASD (Figure 1F), with more pathways coming in/going out from the gray matter between insular cortex and the circular sulcus of the insula in the control (Figure 1E) compared to the ASD sample (Figure 1F). Short tractography pathways were more coherent in the control (Figure 1G), and more varied lengths and directionality with less coherency was observed in ASD (Figure 1H).

### Clinical and *ex vivo* MRI—SVZ pathways

To match the age range with that of epigenetic data, we used all available diffusion tractography data from 15 to 16YO subjects (Figure 4). The data were from five 16YO and one 15 YO male patients with ASD, and six 16YO male neurologically healthy subjects. Among the six patients with ASD, tractography pathways between the SVZ and the insular cortex were found in three patients, while such pathways were not found in any of the six controls.

Tractography pathways from the entire SVZ are shown (Figure 4; yellow) in patients with ASD (15–16 years old) and controls (16 years old). Mid-sagittal slices (right column), lateral sagittal slices at the level of the insula (middle column), and posterior oblique views with mid-sagittal slices and coronal slices at the level of the insula (left column) are shown. Pathways between the posterior SVZ and the insula were absent or fewer in controls compared to ASD patients.

Next, we specifically placed our regions of interest in our clinical data in a dorsal SVZ where we sampled our *ex vivo* specimens for the WGBS analyses. Tractography pathways associated with the dorsal SVZ in 16YO male controls (Figures 2A, B) and 16YO male patients diagnosed with ASD (Figures 2C, D). We examined *in vivo* diffusion tractography in coronal slabs at the level of the insula and thalamus. Tractography pathways were more sparse and fewer in ASD (Figures 2C, D) compared to those found in controls (Figures 2A, B). The same analyses were performed in *ex vivo* brains (Figure 3). Tractography pathways associated with the dorsal SVZ in control brains [Figures 3A–C (#6032; 4 years and 51 days old) and Figures 3G–I (#5282; 2 years 308 days old)] and brains diagnosed with ASD [Figures 3D–F (#M4021M; 3 years 114 days old) and Figures 3J–L (#5308; 4 years 183 days old)]. Yellow spheres (4 mm radius) in each brain image were created in the dorsal SVZ as regions of interest at the coronal level of around the center of the thalamus. Tractography pathways without the spheres are shown in Figures 3C, F, I, L. Tractography pathways were more sparse and fewer in ASD (Figures 3E, F, K, L) compared to those found in control (Figures 3B, C, H, I).

### IPA analysis of insular cortex samples

Postmortem samples of the insular cortex, as described above (Control #5282, ASD #5308), were further analyzed molecularly. To determine whether neurons in this area exhibit a methylomic signature of ASD, we evaluated DNA methylation using WGBS. Similar to our previous report (Corley et al., 2019), we then filtered this list based on absolute mean differences in methylation of 10% or more between the ASD and control groups (δ ≥ |0.10|), which yielded 5585 CpGs that we define as differentially methylated sites associated with ASD. To further explore those 5,585 differentially methylated sites, we used Ingenuity Pathway Analysis (IPA) software to predict biological functions and gene regulatory networks and diseases associated with these DML. As observed in Supplementary Table 1, in the category Diseases and Disorders we had two networks interesting to ASD: Developmental Disorder, with a *p*-value range of 1.67E^–02^–1.67E^–18^ and 62 genes included; and Neurological Disease, with a *p*-value range of 1.67E^–02^–6.40E^–16^ and 118 genes included. In the Physiological System Development and Function category, a noteworthy network was Nervous System Development and Function with a *p*-value range of 1.76E^–02^–6.33E^–09^ and 125 genes included. The list of all genes with methylation changes in each of these networks can be observed in Supplementary Text 1.

**FIGURE 6:**
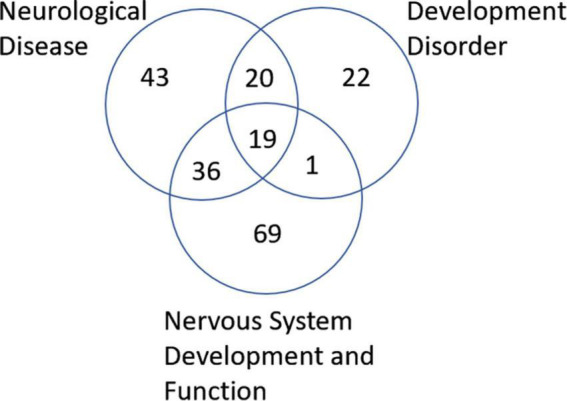
Venn diagram of genes associated with neurological disease, developmental disorder, and nervous system development and function found to be altered in ASD according to IPA analysis. A total of 19 genes were found to overlap all three groups.

**FIGURE 7:**
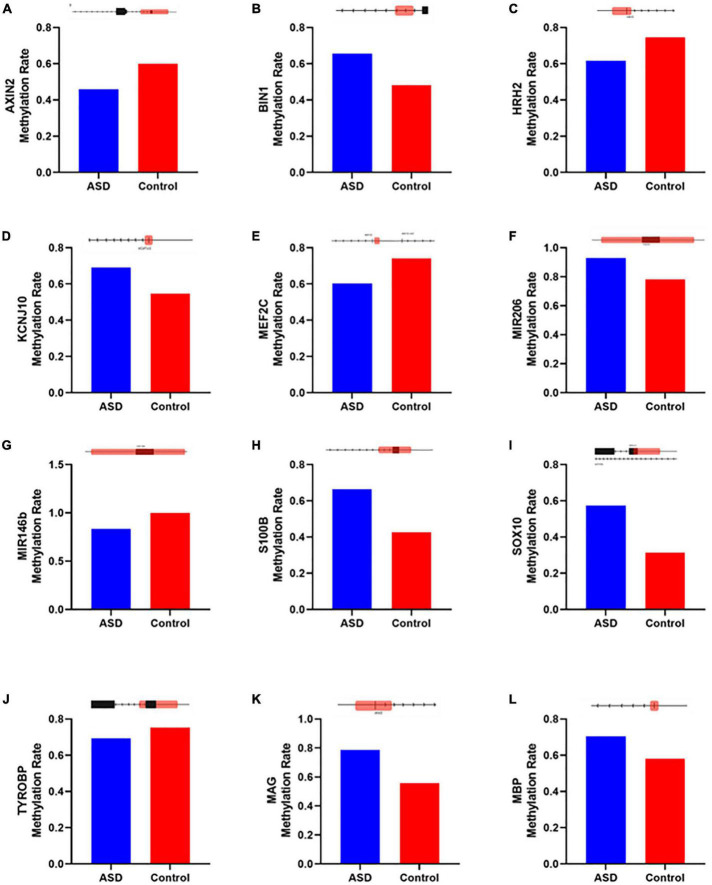
Genes altered in ASD samples according to IPA that overlap with neurological disease, developmental disorder, and nervous system function and development. Methylation levels were compared between ASD and Control insular cortex samples for genes **(A)** AXIN2, **(B)** BIN1, **(C)** HRH2, **(D)** KCNJ10, **(E)** MEF2C, **(F)** miR-206, **(G)** miR-146b, **(H)** S100B, **(I)** SOX10, **(J)** TYROBP, **(K)** MAG, and **(L)** MBP. Diagrams above each bar graph show a linear depiction of the associated gene from 5′ to 3′ with the methylation site highlighted in red (1099 bp). Diagrams are not to scale. Reference genome RGCh38/hg38.

Next, we wanted to examine some of these genes with changes in their methylation. First, we checked which genes were overlapping in between the 3 most enriched networks that could relate to ASD: Developmental Disorder, Neurological Disease, and Nervous System Development and Function. This approach gave us 19 genes to investigate in more detail (Figure 6). Comparison of the methylation levels of each of these genes demonstrates varying levels of altered methylation between Control and ASD samples (Figure 7 and Supplementary Figure 1). While research into each of these genes indicates important roles in the normal function and development of neuronal systems, further research is needed to determine their exact role (or lack thereof) in ASD specifically.

### DNMT family expression levels in SVZ

**FIGURE 8:**
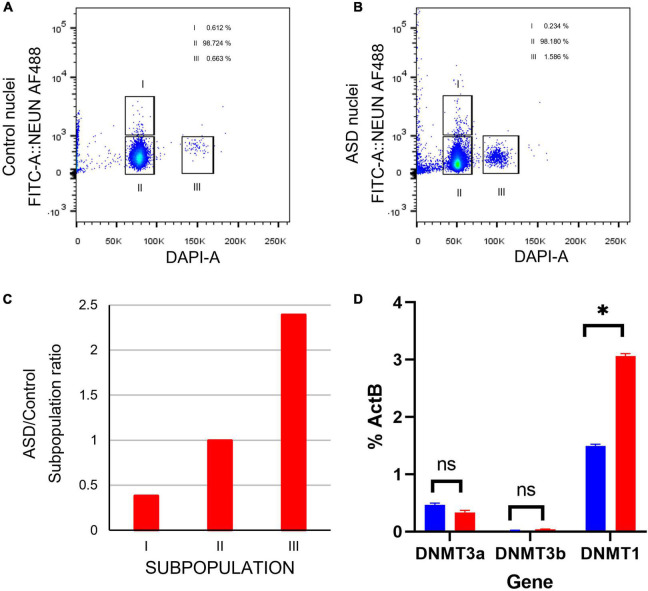
Cell sorting by flow cytometry indicates a higher ratio of proliferating cells in postmortem SVZ tissue of ASD sample. **(A)** Fluorescence Activated Cell Sorting (FACS) of nuclei from in postmortem SVZ brain tissue of control (male, 42 years old) and **(B)** ASD (male, 43 years old) patients’ samples. Nuclei were purified by sucrose gradient and stained with Alexa488NeuN antibody and DAPI. Gates indicated as I select nuclei from mature neurons (Alexa488NeuN+DAPI+) and II select nuclei from glial cells (Alexa488NeuN-DAPI+), nuclei from these gates were sorted for expression analysis. Gate labeled as III select 2X DAPI+ Alexa488NeuN- glial dividing cells. **(C)** ASD/Control ratio of nuclei from the three cell types. **(D)** qPCR expression analysis of cell stage gene markers in postmortem SVZ brain tissue of control (male, 42 years old) and ASD (male, 43 years old) patients’ samples *p < 0.05.

RNA isolated from whole tissue SVZ samples was tested through qPCR for expression levels of DNA methyltransferase 1, 3a, and 3b (DNMT1, DNMT3a, DNMT3b) due to their roles in epigenetic regulation. DNMT1 was found to be significantly elevated in ASD samples (*p* = 0.0385, Figure 8C). This pattern of elevated DNMT1 expression levels appears to be consistent across most ages tested (Figure 8C). No significant changes were observed between control and ASD groups for DNMT3a or DNMT3b; nor was any pattern observed based on age (Figure 8C).

### Cell maturation and proliferation markers in SVZ

**FIGURE 9:**
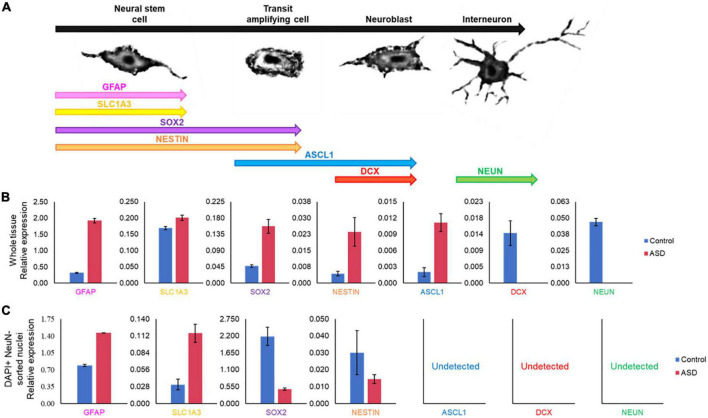
Gene expression pattern in SVZ of ASD and control patients’ samples demonstrates divergent populations of cell stages. **(A)** SVZ cell types and specific cell stage gene markers. **(B)** qPCR expression analysis of cell stage gene markers from the same postmortem SVZ brain tissue samples as in Figure 8. **(C)** qPCR expression analysis of cell stage gene markers in sorted nuclei from Figure 8.

Certain genes are differentially expressed depending on the stage of cell maturation. Glial fibrillary acidic protein (GFAP), which plays an important role in neuronal mitosis (Tardy et al., 1989), and Excitatory amino acid transporter 1 (SLC1A3), which controls glutamate signaling (Pajarillo et al., 2019), are typically expressed in NSCs. SRY-box 2 (SOX2), which is responsible for stem-cell maintenance (Rizzino, 2009), and neuroepithelial stem cell protein (NESTIN) (Michalczyk and Ziman, 2005), an indicator of neuronal cell division, are expressed in NSCs and transit amplifying cells. Achaete-scute homolog 1 (ASCL1), which is important for neuronal differentiation (Nouruzi et al., 2022), is expressed in transit amplifying cells and neuroblasts, while Doublecortin (DCX) is expressed exclusively in neuroblasts and is important for neuronal migration (Brown et al., 2003). Mature interneurons can be distinguished by expression of Neuronal Nuclear Antigen (NeuN) (Mullen et al., 1992), which is widely used as a neuronal marker. These genes together are used to identify the life stages of neurons present, providing information on maturation and development (Figure 9A). Therefore, using qPCR we measured the expression levels of each gene in postmortem SVZ brain tissue of Control (male, 42 years old) and ASD (male, 43 years old) patients’ samples. Whole tissue expression levels indicated elevated levels of GFAP, SLC1A3, SOX2, NESTIN, and ASCL1 in the ASD sample, but little to no expression of DCX and NEUN (Figure 9B). Nuclei were sorted using FACS based on NeuN and DAPI signaling (Supplementary Figure 2). Three distinct populations were observed: gate I indicates mature neurons positive for NeuN and DAPI; gate II indicates glial cells negative for NeuN; and gate III indicates proliferating glial cells that stain with 2X DAPI but are negative for NeuN (Figures 8A, B). The number of cells in each gate for the ASD and Control samples shows a higher ratio of dividing glial cells in ASD compared to control (Figure 8C). To further analyze these differences, we performed the same qPCR as above on the isolated 2X DAPI + NeuN- sorted nuclei (Figure 9C). GFAP and SLC1A3 was found to be elevated in ASD relative to Control, while SOX2 and NESTIN was found to be expressed in higher levels in Control. Indicators of neuroblasts and interneurons—ASCL1, DCX, and NEUN—were not detected in either sample, indicating the cell sorting selected for earlier progenitor cells. We also further tested for markers of “stemness,” differentiation, and proliferation (Supplementary Figure 3). In the whole tissue samples, ASD had consistently higher levels of all markers we tested for, but in the sorted samples only LIFR and SEPP1 remained consistently higher. RBP1 in fact was elevated compared to ASD in the Control sorted sample. FAM107A and EP300, though elevated in the whole tissue ASD sample, were expressed at similar levels in the sorted samples.

## Discussion

**FIGURE 10:**
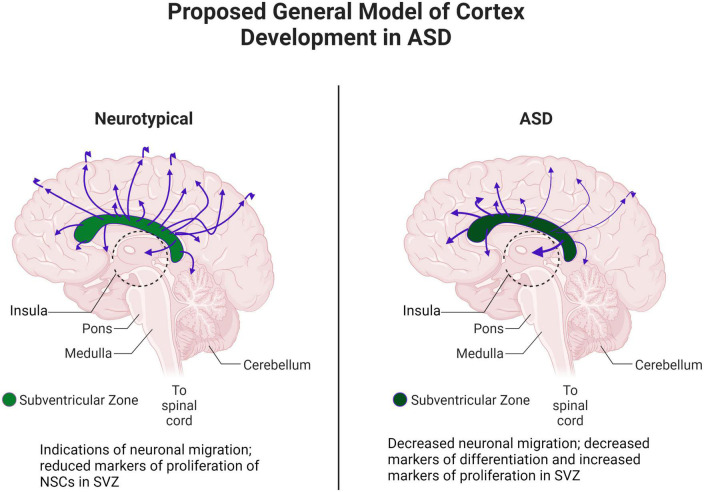
Proposed general model of cortex development in ASD. Adapted from “Distribution of Histamine and Serotonin Neurotransmitters in the Human Brain” by BioRender.com (2022). Retrieved from https://app.biorender.com/biorender-templates.

In this study, we combined our structural/diffusion MRI, WGBS, and gene expression data. In particular, MRI and WGBS were performed on the same postmortem brain samples and suggested interesting patterns of epigenetic dysregulation and dysfunction of proliferation, maturation, and migration in ASD (Figure 10). These patterns were highly conserved in ASD brains, indicating potential defects in the NSC population in the SVZ that may contribute to the altered architecture observed in the insula. Altogether, our preliminary data in this case study suggest that the SVZ and by extension the insular cortex, through control of neuronal migration, maturation, and proliferation, are especially important brain regions that are altered in ASD.

### MRI assessment of insular cortex and white matter pathways in ASD

The insular cortex has been associated with interoception (sensitivity to stimuli originating inside of the body), emotion, and awareness, as well as attention, executive functioning, and decision-making in typically developing subjects (Craig A. D. B., 1996, Craig, 2009a,b; Craig A. D., 2010; Craig A. D. B., 2010; Shelley and Trimble, 2004; Kurth et al., 2010; Nieuwenhuys, 2012; Droutman et al., 2015; Ghaziri et al., 2017; Gogolla, 2017; Uddin et al., 2017). We found changes in the insular cortex in ASD in both clinical and *ex vivo* MRI studies.

Although we previously found increased mean CT of the right insular cortex in Rett syndrome (Shiohama et al., 2019), which is a congenital disorder characterized by autistic features, the current study found no difference in the mean CT between ASD and control groups. However, we found increased variability of the insular CT in ASD patients. The CT variability could be explained by some possibilities such as migrated neurons distributed unevenly in the cortex, uneven regional intra-cortical and/or WM myelination, and synaptic and/or local axonal pruning dysfunction. In our past study (Levman et al., 2019), we also found abnormalities of this measurement in several brain regions in ASD, which may be explained as disorganized cortical development in the ASD population that can be detected by structural MRI.

Decreased long-range pathways between the insular cortex and a ventral part of the sensorimotor cortex, including the somatosensory area, were also found in this study in *ex vivo* ASD specimens. There are almost no diffusion MRI tractography studies on the insular cortex in ASD, but studies using resting-state functional MRI (rs-fMRI) connectivity, although still controversial (Di Martino et al., 2011; Xu et al., 2018), have reported insular hypoconnectivity in ASD (Anteraper et al., 2020; Shi et al., 2020; Zhao et al., 2022), including decreased or altered functional connectivity between the insular and somatosensory cortices (Wang et al., 2017; Zhao et al., 2022). Impaired social cognition is one of the most prominent symptoms of ASD and may be associated with altered integration of the information and/or atypical connectivity of the insula (Uddin and Menon, 2009; Odriozola et al., 2016; Yamada et al., 2016; d’Albis et al., 2018; Pereira et al., 2019).

Although ASD is generally associated with cognitive and sensory difficulties, distinctive motor abnormalities such as repetitive behaviors, atypical gait (Esposito et al., 2011; Nayate et al., 2012), and dyspraxia (Ming et al., 2007) are also sometimes associated with ASD. Studies on motor functions revealed impairments in connections related to motor cortices (Brito et al., 2009; Shukla et al., 2011). The posterior insula has been reported to have connections with the primary and supplementary motor areas (Lu et al., 2016; Ghaziri et al., 2017). Carper et al. (2015) reported functional overconnectivity in corticospinal pathways in ASD, specifically in dorsal areas of the primary motor cortex. Given that coronal slabs were examined in this study, identifying the terminal regions of tractography was not always straightforward. Therefore, we mentioned the regions where tractography started/terminated as the sensorimotor/somatosensory cortices.

Although local intra-insular pathways were not clearly different between the ASD and control specimens in this study, rs-fMRI studies have found reduced intra-insular connectivity in ASD (Ebisch et al., 2011; Jiang et al., 2015). Another study found reduced intrainsular WM integrity (increased mean diffusivity) in ASD (Failla et al., 2017). Although our results were not clear, it would be possible to find statistical abnormalities in ASD with a greater number of specimens in a future study. In addition, our specimens were coronal slabs that are available at the NeuroBioBank, so only within-coronal pathways were examined in this study. Similar to the long-range pathways above, with whole hemispheres or whole brains, it would be possible to assess the entire insular pathways in the future.

### MRI assessment of the subventricular zone and its pathways in ASD

Our results showed that tractography pathways from the dorsal SVZ to the sensorimotor/somatosensory cortices were reduced, while the lateral ventral SVZ pathways to the insular cortex were increased in ASD. Together with our CT variability findings and tractography results linked to axonal pathways, one could hypothesize that abnormally increased neuronal migration from the ventral SVZ to the insula (Meyer, 2018) may result in disorganized neuronal organization in the insular cortex measured by CT variability, and reduced axonal pathways from the insular cortex. For example, heterotopias, increased regional density of neurons at the gray-WM junction, and focal cortical dysplasias are often observed in ASD (Casanova et al., 2020), which could be associated with CT variability. In addition, abnormally reduced neuronal migration from the dorsal SVZ to the sensorimotor/somatosensory cortices (Jurkowski et al., 2020) might affect reduced connectivity from the sensorimotor/somatosensory cortices to the insular cortex. These together could be a basis for behavioral and cognitive dysfunctions associated with the insular cortex.

### Differentially expressed genes are associated with neurological disease, disorders, and nervous system development and function

IPA analysis of WGBS data from dorsal insula brain samples identified differential methylation patterns in components of three potentially important networks in ASD: neurological disease, developmental disorders, and nervous system development and function (Supplementary Table 1). Of these three networks, we identified 19 genes that are involved or predicted to be involved in all three networks (Figure 6, the raw data for these genes can be seen in Supplementary Table 2).

Of these 19 genes, 8 have previously been identified as contributors to an ASD-like phenotype. AXIN2 (Figure 7A) has been described as related to language impairments in patients in the autism spectrum through the Wnt signaling pathway (Benítez-Burraco et al., 2016). BIN1 (Figure 7B) is known to contribute to the select impairment of spatial learning and memory (De Rossi et al., 2020), possibly contributing to learning disabilities observed in ASD (O’Brien and Pearson, 2004). HRH2 (Figure 7C) is known to inhibit nerve cells and block long-lasting after hyperpolarization and accommodation of firing in cortical and thalamic neurons (Brown et al., 1995). HRH2 was found to have high expression in ASD cases, including data that receptor antagonists could be used for behavioral and sleep disturbance improvement in children and adolescents with ASD (Rossi et al., 1999; Linday et al., 2001; Wright et al., 2017). KCNJ10 (Figure 7D) mutations contribute to ASD with seizures and intellectual disability (Sicca et al., 2011). Also, the dysfunction of the KCNJ10 protein, Kir4.1, is associated with a number of neuronal phenotypes in several syndromic or non-syndromic neurodevelopmental disorders presenting with broad clinical manifestations and encompassing movement disorders and intellectual disability, besides ASD and seizures (Sicca et al., 2016). The MEF2C (Figure 7E) gene plays an important role in activity-dependent synaptic elimination, possibly contributing to the excitatory/inhibitory synaptic ratio in ASD (Zhang and Zhao, 2022). S100B (Figure 7H) has been shown to affect the synaptic SHANK2 and SHANK3 levels in a zinc-dependent manner, notably early in neuronal development. Mice exposed to high S100B levels *in utero* similarly show reduced levels of free zinc and SHANK2 in the brain. In terms of behavior, these mice display hyperactivity, increased stereotypic and abnormal social behaviors, and cognitive impairment (Daini et al., 2021). Reduced expression of SOX10 (Figure 7I), a transcription factor, was observed in the limbic system including the hippocampus, retrosplenial cortex, and para hippocampal gyrus of mice related to autism (Zhang et al., 2020). Differential expression of a transmembrane immune signaling adaptor, TYROBP (Figure 7J), was observed in the prefrontal cortex (PFC) of postmortem brain tissue from children with ASD as compared to controls (Edmonson et al., 2014). Beyond that, mutations in this gene lead to Nasu–Hakola disease, a rare autosomal recessive disorder characterized by bone abnormalities and adult-onset neuropsychiatric features, such as social disinhibition, distractibility, and lack of appropriate emotionality, very similar to common ASD symptoms (Paloneva et al., 2002). Also, although myelin associated glycoprotein (MAG) and myelin basic protein (MBP) (Figures 7K, L) have no data specific to ASD, higher levels of anti-MAG and anti-MBP antibodies were detected in ASD patients compared to controls, indicating a role in neuroinflammation, as these antibodies have been shown to react against neuronal tissue (Zou et al., 2020).

Additionally, we identified CpGs over two miRNAs that exhibited significantly different levels of methylation in ASD; both miRNAs have been previously implicated in ASD. First, the family of miR-206 (Figure 7F) has been described to confer susceptibility to ASD (Toma et al., 2015), most likely for its ability to regulate BDNF, a key regulator of synaptic plasticity (Lee et al., 2012). Second, miR-146b (Figure 7G) was reported to be overexpressed in the brain of ASD patients early in childhood. *In vitro* analyses of NSCs suggested that miR-146a contributes to the regulation of balancing cell-cycle exit and re-entry of neural progenitors and commitment to neural differentiation pathways (Nguyen et al., 2018). Furthermore, miR-146 expression contributed to neuroinflammation in the brain of ASD subjects, having a role in immune system regulation (Tonacci et al., 2019). Recapitulating abnormal miR-146 expression in mouse primary cell cultures led to impaired neuronal dendritic arborization—producing shriveled dendritic trees with branching points at more proximal levels compared to controls, mirroring the defective neural connectivity typical of ASD—and to increased astrocyte glutamate uptake capacities (Nguyen et al., 2016). The remaining seven genes identified by IPA have not been previously identified in ASD (Supplementary Figure 1) and warrant further investigation.

The altered methylation status of these genes in the insular cortex of ASD brains, combined with past findings highlight these genes as of interest in further ASD research. However, as these results depend solely on data obtained from one pair of samples in this case study, further analyses are required to determine their significance. Despite this limitation, the alteration of genes associated with these networks adds support to our hypothesis that the ASD phenotype is a result of early alterations to the developmental trajectory of cells that comprise the SVZ-cortical axis.

### ASD phenotype associated with increased markers of proliferation

In examining two samples of the postmortem SVZ *via* flow cytometry, we observed three distinct populations: neuronal cells (I), glial cells and NSCs (II), and proliferating cells (III). When comparing the ratios of these populations between ASD and unaffected, control samples, we found that the ASD sample had an increased ratio of proliferating cells present than that of the control (Figure 8C). We tested these samples for established cell maturation markers (Figure 9A) and compared any changes between the whole tissue samples (Figure 9B) and the sorted proliferating cells (Figure 9C). The status of population III as proliferating neurons is supported by our data that these cells expressed elevated levels of early cell cycle stage genes GFAP and SLC1A3, while makers of later stages, ASCL1, DCX, and NEUN, were completely absent (Figure 9C). Additionally, expression levels of gene markers for earlier stages of development, which suggest the presence of NSCs or transit amplifying cells, were elevated in ASD compared to that of the control, while the expression of genes associated with more mature neurons, such as interneurons or neuroblasts, were decreased (Figure 9C). This indicated that this population of cells tended to have an increased “stemness” phenotype in ASD. This trend was not consistent in the whole tissue sample tested, likely masked by other cell types, yet the ASD sample exhibited higher levels of earlier cell stage-specific markers than that of the control (Figure 9B). The whole tissue ASD sample also exhibited elevated levels of ASCL1, a marker of transit amplifying cells and neuroblasts, whose expression was not detected in population III, similar to later cell stage markers DCX and NeuN (Figure 9B).

We performed additional testing on these same samples for markers of “stemness,” differentiation, and migration. Our results indicate increased proliferation and increased population of early cell cycle cells in ASD, with decreased indications of migration and differentiation signals, providing further support for our observations of increased proliferation and aberrant migration and differentiation in the SVZ of ASD (Supplementary Figure 3).

### Expression of epigenetic control gene in ASD indicates increased proliferation in SVZ

DNMT1 is known to play a key role in mammalian development and is essential for the maintenance and control of methylation patterns during and following DNA replication (Qin et al., 2011; Chen and Zhang, 2020). Aberrant expression of DNMT1 has been associated with altered proliferation, differentiation, and migration patterns in the developing and adult brain (Symmank and Zimmer, 2017). The observation of a change in expression of DMNT1 but not its related DNA methyltransferase genes DNMT3a or DNMT3b (Figure 8D) can be explained because the DNMT3 family is most often expressed during embryogenesis and gametogenesis while DNMT1 is more ubiquitous (Okano et al., 1999; Qin et al., 2011). Previous studies have demonstrated that DNMT1 promotes survival of proliferating cells in the dentate gyrus (Noguchi et al., 2015) (one of two sites with demonstrated adult neurogenesis) and plays an essential role in migration, morphology, and successful pruning of cortical neurons (Pensold et al., 2017; Symmank and Zimmer, 2017). DNMT1, however, is not essential for the survival of post-mitotic cells, implying that its essential role in the promotion of neuronal survival occurs before or during mitosis (Noguchi et al., 2015). Thus, in these cases, the increased expression of DNMT1 in ASD relative to control SVZ samples may associated with the increased cell proliferation observed.

The combination of our MRI studies and expression analysis of the SVZ and insular cortex suggests that an underlying cause of the ASD phenotype is decreased or mis-targeted migration of neuronal precursors during development, and an increased inclination toward maintaining populations of undifferentiated proliferating cells in the SVZ in adolescence and adulthood. While the mechanism by which this phenotype occurs is not clear, our study demonstrates a way in which the structure of the cortex is altered during and after development in ASD, suggesting an underlying cause of the observed socio-emotional, sensorimotor and auditory processing, and executive function aberrations in ASD (Uddin et al., 2017). Our data, although preliminarily, suggest a promising explanation for many aspects of the ASD phenotype as well as an associated aberrant brain structure. Further study with larger datasets will be necessary to determine the correlation between structural and expression changes and ASD.

## Data availability statement

The original contributions presented in this study are included in the article/Supplementary material, further inquiries can be directed to the corresponding authors.

## Ethics statement

The studies involving human participants were reviewed and approved by the Boston Children’s Hospital Human Research Committee. Written informed consent to participate in this study was provided by the participants’ legal guardian/next of kin.

## Author contributions

ET, NA, RP, AO, AK, JL, and AM wrote the manuscript. ET, NA, RP, AO, AK, BV, EE, JL, KT, NV-M, TM, RL, and AM obtained the data. ET, NA, RP, AO, JL, NB, KT, NV-M, and AM analyzed the data. All authors contributed to the article and approved the submitted version.

## Footnotes

1
http://trackvis.org
