# Supplementary material for: Integration of structural MRI and epigenetic analyses hint at linked cellular defects of the subventricular zone and insular cortex in autism: Findings from a case study
Source: Front Neurosci. 2023 Feb 3;16:1023665. doi: 10.3389/fnins.2022.1023665 (PMC9935943; doi:10.3389/fnins.2022.1023665)
Supplement: Supplementary file 1 [file Data_Sheet_1.pdf]

Supplemental Table 1: Gene regulatory networks predicted to be associated with ASD according to analysis with IPA based on two samples from postmortem insular cortex (methylation changes >10%).

**Top Canonical Pathways**	**p-value**	**Overlap/Molecules**	
Graft-versus-Host Disease Signaling	2.06E-09	7.2%	32/446
OX40 Signaling Pathway	3.17E-09	6.9%	33/479
Autoimmune Thyroid Disease Signaling	3.18E-09	7.0%	32/454
ICOS-ICOSL Signaling in T Helper Cells	3.61E-09	6.7%	34/507
Th17 Activation Pathway	4.10E-09	6.8%	33/484

**Diseases and Disorders**	**p-value range**	**Molecules**
Developmental Disorder	1.67E-02 - 1.67E-18	62
Hereditary Disorder	1.36E-02 - 1.67E-18	75
Organismal Injury and Abnormalities	1.78E-02 - 1.67E-18	236
Skeletal and Muscular Disorders	1.14E-02 - 1.67E-18	86
Neurological Disease	1.67E-02 - 6.40E-16	118

**Molecular and Cellular Functions**	**p-value range**	**Molecules**
Gene Expression	9.80E-38 - 9.80E-38	51
Cellular Development	1.63E-02 - 6.33E-09	156
Cell Cycle	1.76E-02 - 1.10E-06	36
Cellular Movement	1.69E-02 - 1.10E-06	125

**Physiological System Development and Function**	**p-value range**	**Molecules**
Skeletal and Muscular System Development and Function	1.40E-02 - 7.21E-10	63
Tissue Morphology	1.52E-02 - 7.21E-10	88
Nervous System Development and Function	1.76E-02 - 6.33E-09	125
Tissue Development	1.76E-02 - 6.33E-09	107
Organismal Development	1.76E-02 - 6.37E-06	98

Supplemental Table 2: Results and locations of differential methylation changes of 19 genes identified via IPA and measured by WGBS

Ensembl ID	symbol	entrezID	Chromosome	Start	End	ASD	Control	Differential (ASD/Control)
ENSG00000168646	AXIN2	8313	chr17	65561148	65563147	0.191301	0.192738	-0.00929
ENSG00000136717	BIN1	274	chr2	1.27E+08	1.27E+08	0.080848	0.141593	-0.06308
ENSG00000113749	HRH2	3274	chr5	1.76E+08	1.76E+08	0.137201	0.176299	-0.03549
ENSG00000177807	KCNJ10	3766	chr1	1.60E+08	1.60E+08	0.08416	0.165777	-0.0622
ENSG00000081189	MEF2C	4208	chr5	88903606	88905605	0.985294	NA	NA
ENSG00000207604	MIR206	406989	chr6	52142831	52144830	0.731786	0.845487	-0.12782
ENSG00000202569	MIR146B	574447	chr10	1.02E+08	1.02E+08	0.494968	0.584606	0.015443
ENSG00000160307	S100B	6285	chr21	46604709	46606708	0.314422	0.509859	-0.19544
ENSG00000100146	SOX10	6663	chr22	37986923	37988922	0.233938	0.541982	-0.33385
ENSG00000011600	TYROBP	7305	chr19	35907796	35909795	0.774951	0.751912	0.023039
ENSG00000105695	MAG	4099	chr19	35290625	35292624	0.150321	0.446214	-0.29632
ENSG00000197971	MBP	4155	chr18	77133184	77135183	0.610896	0.755853	-0.11467
ENSG00000108556	CHRNE	1145	chr17	4902575	4904574	0.712583	0.646309	0.066275
ENSG00000207975	MIR181B1	406955	chr1	1.99E+08	1.99E+08	0.53687	0.878064	-0.12792
ENSG00000275402	MIR155	406947	chr21	25572480	25574479	0.693948	0.52381	-0.04762
ENSG00000208024	MIR199A2	406977	chr1	1.72E+08	1.72E+08	0.838358	0.856434	-0.00453
ENSG00000168314	MOBP	4336	chr3	39465698	39467697	0.725192	0.84742	-0.15953
ENSG00000170989	S1PR1	1901	chr1	1.01E+08	1.01E+08	0.035802	0.072389	-0.03736
ENSG00000174607	UGT8	7368	chr4	1.15E+08	1.15E+08	0.020546	0.036062	-0.01951
