# Supplementary material for: Integration of structural MRI and epigenetic analyses hint at linked cellular defects of the subventricular zone and insular cortex in autism: Findings from a case study
Source: Front Neurosci. 2023 Feb 3;16:1023665. doi: 10.3389/fnins.2022.1023665 (PMC9935943; doi:10.3389/fnins.2022.1023665)
Supplement: Supplementary file 2 [file Data_Sheet_2.pdf]

Supplemental Text 1: Genes involved in predicted networks (Supplemental Table 1), grouped by associated function by IPA.

Neurological Disease genes (118):
AGO2, AXIN2, BIN1, BTBD8, CA2, CACNA1C, CAMK2A, CBS/CBSL, CD9, CD19, CD68, CELF2, CHRM1, CHRNA7, CHRNE, CNOT3, CNP, CORT, CRYAB, CTSB, DBI, DUSP10, DYNLT4, EEF2K, EPHB6, GNG3, GPR17, GPR37, HCST, HLA-DQB1, HLA-DRB1 ,HRH2 , HSPB3, IL17C, KCNJ10, KLF6, MAG, MAML1, MAPK3, MAPT, MBP, MEF2C, MIR3185, MIR4740, mir-1, mir-7, mir-15, mir-21, mir-25, mir-26, mir-29, mir-30, mir-33, mir-34, mir-96, mir-103, mir-124, mir-128, mir-130, mir-142, mir-145, mir-146, mir-148, mir-153, mir-154, mir-155, mir-181, mir-182, mir-183, mir-199, mir-204, mir-216, mir-326, mir-328, mir-329, mir-342, mir-346, mir-368, mir-455, mir-515, mir-548, mir-550, mir-551, mir-648, mir-650, mir-657,mir-873, mir1306, MOBP, MYRF, NAT8L, NAXE, NCF1, NPPA, NPPB, OLFM1, OLFML3, OPALIN, OR4C46, OR5M1, ORM2, POLR2F, PROCR, PTEN, QKI, S100B, S1PR1, SLC31A2, SOX10, TAMALIN, TF, THY1, TMEM204, TNFRSF4, TXNIP, TYROBP,UGT8, VTRNA2-1

Developmental Disorder Genes (62):
AXIN2, BGLAP, BIN1, CELF2, CHRNE, HLA-DQB1, HLA-DRB1, HRH2, KCNJ10, LGALS7/LGALS7B, MAB21L1, MAG, MAML1, MBP, MEF2C, mir-1, mir-103, mir-1225, mir-128, mir-130, mir-1306, mir-145, mir-146, mir-148, mir-15, mir-154, mir-155, mir-181, mir-199, mir-21, mir-216, mir-25, mir-29, mir-299, mir-30, mir-302, mir-329, mir-34, mir-345, mir-346, mir-365, mir-368, mir-379, mir-548, mir-648, mir-650, mir-654, mir-7, mir-96, MOBP, MYRF, OLFM1, POLR2F, S100B, S1PR1, SOX10, TFEB, TMEM204, TYROBP, UGT8, UPK3A, XK

Nervous System Development and Function Genes (125):
ADAMTS4, AGO2, AXIN2, BIN1 , CACNA1C, CAMK2A, CBS/CBSL, CD9, CHRM1, CHRNA7, CHRNE, CHST3, CNP, CORT, CRYAB, CTSB, DBI, DUSP10, EEF2K, GAS2L1, GPR17, GPR37, GPR37L1, HLX, HRH2, KCNJ10, LHX2, MAG, MAPK3, MAPK8IP2, MAPT, MBP, MEF2C, mir-1, mir-15, mir-21, mir-26, mir-29, mir-30, mir-34, mir-96, mir-124, mir-138, mir-146, mir-155, mir-181, mir-182, mir-183, mir-199, mir-204, mir-219, mir-449, MOBP, MYRF, MYT1, NEUROD6, NPFF, NPS, OPALIN, OR10G9, OR10X1, OR11H4, OR14C36, OR1I1, OR1J4, OR2AT4, OR2L2, OR2M3, OR2M5, OR2T8, OR4A5, OR4A15, OR4A47, OR4C6, OR4C11, OR4C15, OR4C46, OR4K1, OR4K2, OR4N5, OR4S2, OR52B4, OR52E4, OR52N4, OR52R1, OR5AC2, OR5AK2, OR5AN1, OR5AP2, OR5AS1, OR5H2, OR5K2, OR5K3, OR5M1, OR6C2, OR6C68, OR6C76, OR6Y1, OR7C1, OR7D4, OR8A1, OR8D4, OR8U1, PPP1R15A, PTEN, QKI, RND1, RND2, RNF112, S100B, S1PR1, SLC12A7, SLC45A3, SOX8, SOX10, SRCIN1, TAMALIN, TF, THY1 TYROBP, UGT8, XK, ZBTB18, ZCCHC24, ZIC4
