# Supplementary figures and images for: Integration of structural MRI and epigenetic analyses hint at linked cellular defects of the subventricular zone and insular cortex in autism: Findings from a case study
Source: Front Neurosci. 2023 Feb 3;16:1023665. doi: 10.3389/fnins.2022.1023665 (PMC9935943; doi:10.3389/fnins.2022.1023665)

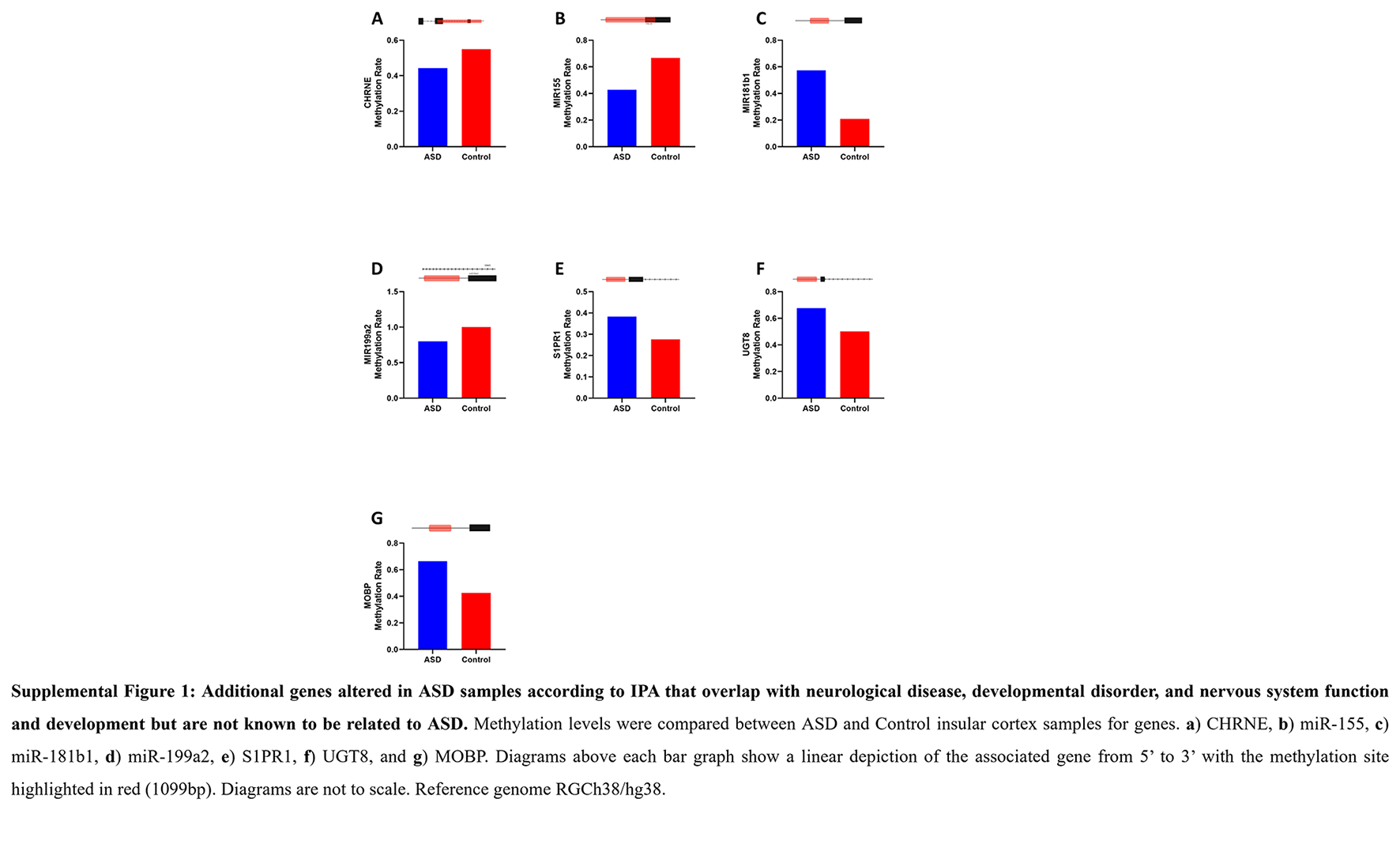

Supplement: Supplementary file 3 [file Image_1.tif]

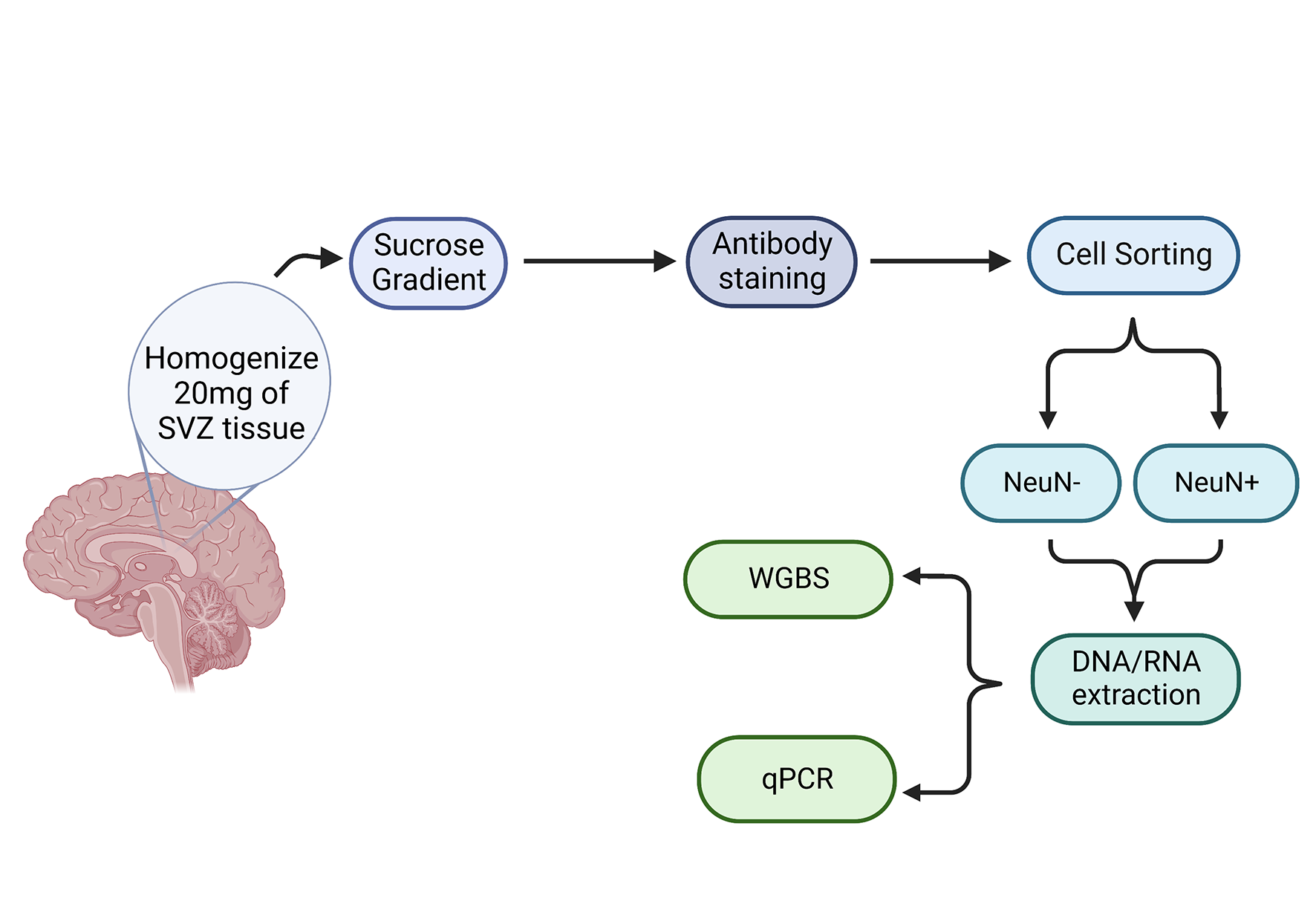

Supplement: Supplementary file 4 [file Image_2.png]

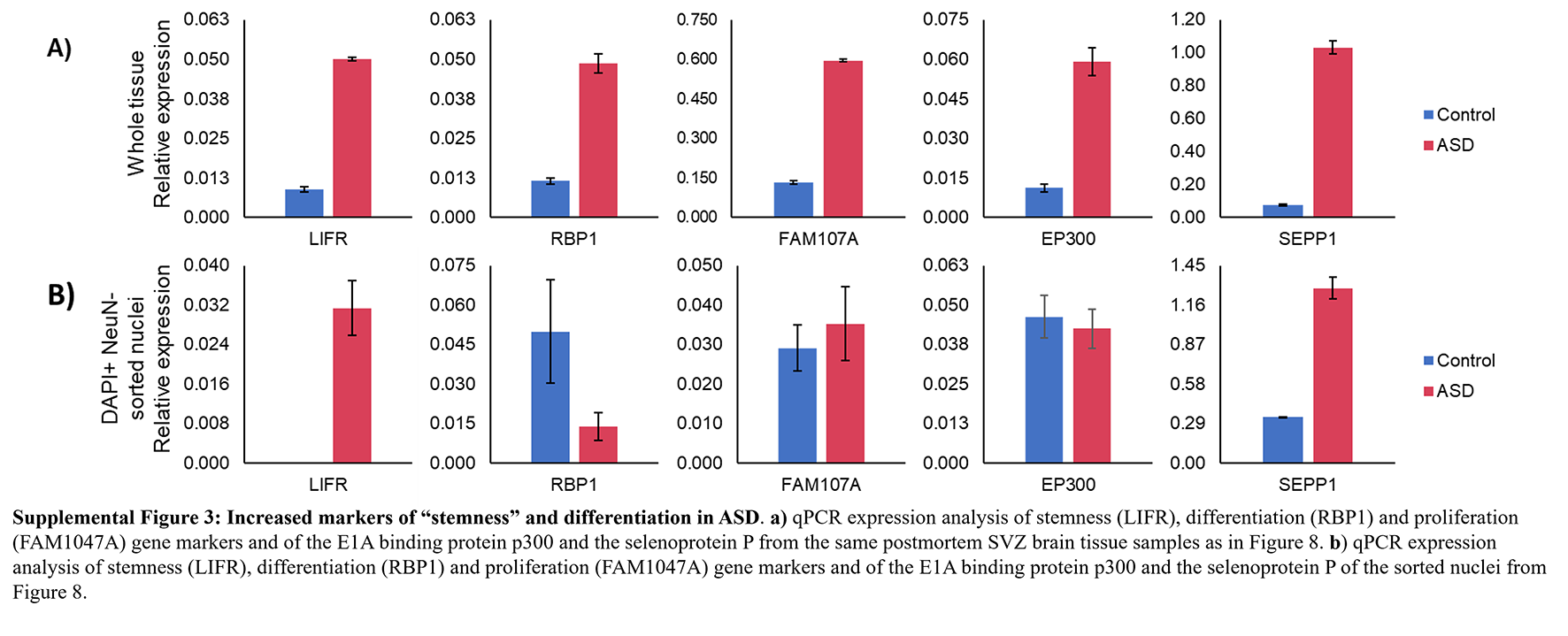

Supplement: Supplementary file 5 [file Image_3.tif]
